# The Elite-Plus stem migrates more than the flanged Charnley stem: A clinical, radiographic, and radiostereometric analysis of 114 patients with an average of 7 years follow-up

**DOI:** 10.3109/17453674.2010.480937

**Published:** 2010-05-21

**Authors:** Thord von Schewelov, Lennart Sanzén, Jack Besjakov, Åke Carlsson

**Affiliations:** Department of Orthopedics, Lund University, Malmö University Hospital, MalmöSweden

## Abstract

**Background and purpose:**

The Charnley Elite-Plus stem was introduced in 1993 as a presumed improvement of the flanged Charnley stem. We started this study in 1996 to investigate the migratory pattern of the Elite-Plus stem.

**Patients and methods:**

We followed 114 patients with osteoarthritis and a primary total hip replacement with the Elite-Plus stem. Mean age at the time of operation was 64 (50–76) years. The mean follow-up time was 6.5 (2–7) years. Radiographs were evaluated with respect to cementing technique, migration, and wear measured by radiostereometry (RSA).

**Results:**

The stem survival was 98% (CI: 96–100) at 7 years and 92% (CI: 86–97) at 10 years. Mean migration of the femoral head was 0.35 mm (SD 0.3) medially, 0.51 mm (SD 0.6) distally, and 1.1 mm (SD 1.8) in the dorsal direction. Mean total point motion was 1.7 mm (SD 1.7). The migration of the stems stabilized after 5 years in the medial and dorsal directions, but continued to subside slightly. Migration along any of the axes was higher if the cementing technique was inferior.

**Interpretation:**

Patients with a Charnley Elite-Plus stem and defects in the cement mantle or other signs of inferior implantation technique should be carefully monitored.

## Introduction

**Figure 1.**
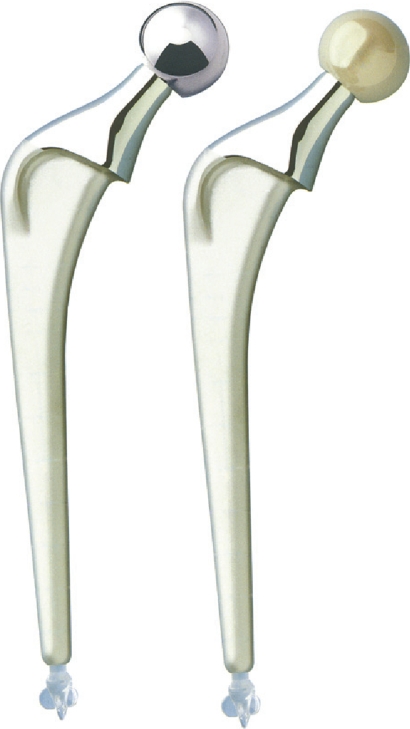
The Elite-Plus stem with centralizer and 22-mm head of steel (left) or zirconium oxide ceramic (right).

The Charnley flanged monobloc stem combined with a cemented socket has a well-documented long-term performance, with a survival rate of approximately 92% at 10 years according to the Swedish Hip Arthroplasty Register (2007). The Charnley Elite-Plus stem was introduced in 1993 as a development of the flanged Charnley stem. The Elite-Plus stem was made more slender distally and offered design alterations such as a modular head with different neck lengths and a distal tip centralizer (Figure 1).

In 1996, we began a randomized radiostereometric (RSA) study with the primary aim of studying the wear pattern for different bearings, and also to investigate the migratory pattern of the Elite-Plus stem. We also wanted to evaluate clinical performance and conventional radiographs. The first part of our study has already been published (von Schewelov et al. 2005). Although the Elite-Plus stem has recently been withdrawn from the market due to the divergent clinical results reported (Norton et al. 2002, Rowsell et al. 2005, Walton et al. 2005, Hauptfleisch et al. 2006), we found it of interest to explore which factors may have contributed to poor outcome. An important issue is whether some or all of the patients who received the Elite-Plus stem may need a more careful follow-up.

## Patients and methods

From 1996 through 1998, 120 consecutive patients with osteoarthritis had a primary total hip replacement using the Elite-Plus stem. The patients were randomized into 4 groups to receive a cup of standard or Hylamer polyethylene combined with either a stainless steel head or a zirconium ceramic head (all implants: DePuy; Johnson & Johnson, Warsaw, IN).

The ethics committee at Lund University approved the study (LU 214-95). 5 patients were excluded because of illness or death unrelated to the hip surgery within the first 2 years. 1 patient was reoperated several times within the first year, due to recurrent dislocations, and was therefore excluded. Thus, 114 patients (62 women) remained to be included in the survival analysis. Their mean age at the time of operation was 64 (50–76) years and their mean body weight was 80 (45–117) kg. Without exception, only one hip per patient was included.

Examinations within 1 week postoperatively and at 3 months and 1, 2, 5, and 7 years included a clinical assessment, and radiographic and radiostereometric analysis. For the clinical evaluation, we used the Harris hip score (HHS) and the Charnley score. The radiographic examination included an AP view of the hip and pelvis and a lateral view of the femur. The radiographs were scrutinized by two of the authors together (ÅC and JB). Change of position, and progressive and non-progressive radiolucent lines were looked for in the femur according to DeLee and Charnley (1976). Loosening was defined as an obvious change of stem position, progressive osteolysis, or progressive radiolucent lines.

Based on their visual impression, the two observers agreed on whether the stem was too small relative to the marrow cavity or not. Quality of the cementing technique was evaluated according to Barrack et al. (1992). In 87 of the 114 patients, the preoperative radiographs could be found and used to template for femoral component size. The template size was then compared to the stem size actually implanted.

12 of the 114 patients had died and 9 had been revised due to aseptic failure between the 2- and 7-year controls, 3 due to stem failure and 6 due to socket failure. These patients were monitored at their last examination before revision or death. 93 patients remained for clinical, radiographic, and RSA examination at 7 years.

For the survival analysis, deaths and stem revisions up to 12 years postoperatively were recorded in May 2008. The Swedish Hip Arthroplasty Register was checked for revisions in other hospitals and deaths were cross-checked with the National Death Registry.

### Prosthesis and operative technique

5 surgeons performed the operations in a clean-air enclosure. Systemic antibiotics and gentamycin-loaded bone cement (Palacos with Gentamycin) were used routinely. The hips were exposed in the supine position through an anterolateral Hardinge approach without trochanteric osteotomy.

In all cases, a cemented Charnley Elite-Plus stem was used (Figure 1). The 22.2-mm modular head was made of high-nitrogen stainless steel or zirconium oxide ceramic. The acetabular component was a Charnley Ogee polyethylene Enduron or Hylamer socket with 40–47 mm outer diameter (all components from DePuy; Johnson & Johnson).

### Radiostereometric analysis

In the proximal femur about 8 tantalum beads, 0.8 mm in diameter, were inserted at the index operation. RSA was performed with the patient in supine position using the uniplanar technique (Selvik 1989). Motion of the head was evaluated relative to the femoral bone. The latter displacement was calculated as transversal, distal, sagittal, and total displacement, i.e. the length of the resulting vector. The radiographs were digitized on a measurement table (Hasselblad Engineering, Gothenburg, Sweden) using linear gauges and a video camera with ×16 magnification. This method was changed for a digital method during the latter part of the study. The images were scanned at 16 bits/300 DPI resolution with an Umax Mirage II scanner (UMAX Techville Inc., Dallas, TX), measured with UmRSA Digital Measure (RSA Biomedical, UmeÅ, Sweden), and analyzed by UmRSA in the same way as with the manual measurements. UmRSA Digital Measure uses least squares fitting of non-linear marker models to estimate the marker center position, a technique that has been shown to maintain or improve the precision compared to manual measurements (Borlin 2000, Borlin et al. 2002, Bragdon et al. 2002). Limits for statistically significant movements over time, with 99% confidence intervals, have been calculated previously using double examinations; this was ± 0.2 mm for the longitudinal and transverse axis and ± 0.5 mm along the sagital axis, and the same for total displacement (önsten et al. 1995). With an experimental setup using a phantom the accuracy has been calculated, using the head in relation to the femoral bone the precision interval was found to be ± 0.14 mm, and the precision 0.03 mm (önsten et al. 2001).

### Statistics

We used unpaired Student’s t-test, Fisher’s exact test, and the Mann-Whitney U-test. Kaplan-Meier survival analysis was used, with aseptic loosening of the stem as endpoint. Wilcoxon matched pairs test was used to calculate prosthetic movement between measurements.

## Results

### Clinical results and revisions

Mean follow-up of all 114 patients was 78 (23–88) months. 8 stems had been revised because of radiographic signs of loosening (Table 1). At the 7-year control or the latest follow up, the mean Harris hip pain score was 41 (SD 6) and the total score was 91 (SD 12).

**Table 1.** Stem revisions

Interval (months)	Case no.	Type of socket ^**a**^	Femoral head	Abnormal socket wear	Findings at revison of stem	Findings at revison of cup	Revision technique (stem)
26	115	Hylamer	Zirconium	Yes	Fixed/Osteolysis	Fixed/Osteolysis	Elite
40	83	Hylamer	Zirconium	Yes	Fixed ^**b**^	Loose/Osteolysis	Elite
70	65	Enduron	Steel	No	Loose	Loose	EX
87	24	Hylamer	Zirconium	Yes	Fixed/Osteolysis	Loose/Osteolysis	EX-B
95	119	Hylamer	Zirconium	Yes	Loose/Osteolysis	Loose/Osteolysis	EX-B
101	41	Hylamer	Zirconium	Yes	Loose/Osteolysis	Loose/Osteolyis	EX-B
115	8	Enduron	Steel	No	Loose/Osteolysis	Not revised	Wagner
112	34	Hylamer	Steel	Yes	Loose/Osteolysis	Loose/Osteolysis	EXB
120	72	Hylamer	Zirconium	Yes	Loose/Osteolysis	Loose/Osteolysis	Corail

Elite: cemented Charnley-Elite Plus; EX: Cemented Exeter stem; EX-B: Exeter stem after bone impaction; Corail: uncemented Corail stem; Wagner: Wagner Revision stem.

Abnormal socket wear implies annual wear of > 0.15 mm.

^**a**^ p = 0.2.

^**b**^ Fixed stem changed “en passant” at revision of cup. Not included in the statistical analysis

### Survival analysis

**Figure 2.**
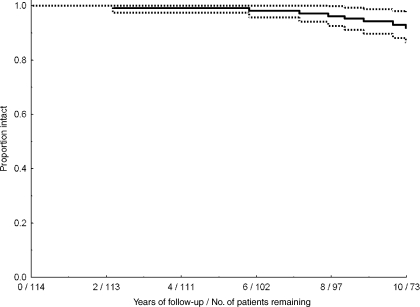
Survival of the 114 Charnley Elite-Plus stems with revision due to aseptic loosening as endpoint (with 95% CI).

The survival rate with aseptic loosening of the stem as endpoint was to 98% (CI 96-100%) at seven years and 92% (CI 86-97%) at 10 years (Figure 2).

### Radiographic analysis

One or more signs of radiographic stem loosening were observed in 15 patients. 8 of these stems with progressive radiographic signs of loosening had been revised and the other 7 patients with minor or non-progressive radiographic signs of loosening are being monitored. 1 additional well-fixed stem with normal appearance on radiographs was changed at cup revision because of joint laxity, making 9 stem revisions in all. This stem was not included in the statistical analyses of revised loose stems (Table 2).

**Table 2.** Migration and stem position, quality of cementing and stem size

	AP view: median migration (min–max)			Side view: mean migration (SD)			Cement grade: mean migration (SD)			Stem size: mean migration (SD)		
	Neutral	Varus	p-value ^**a**^	Neutral	Dorsal	p-value ^**b**^	A	B/C	p-value ^**b**^	Adequate	Small	p-value ^**b**^
Number	88	8		70	28		76	22		72	26	
Transversal (x)	0.26	0.26	0.4	0.34	0.38	0.8	0.20	0.89	0.0003	0.28	0.52	0.16
	(-0.9–2.3)	(0.0–6.6)		(0.9)	(0.7)		(0.4)	(1.4)		(0.5)	(1.3)	
Longitudinal (y)	-0.46	-0.42	0.8	-0.51	-0.52	0.9	-0.41	-0.86	0.0004	-0.50	-0.54	
	(-3.6–0.94)	(-4.17–0.14)		(0.6)	(0.8)		(0.5)	(1.0)		(0.6)	(0.81)	0.78
Sagittal (z)	-0.88	-0.97	0.4	-0.95	-1.6	0.09	-0.92	-1.89	0.02	-0.95	-1.6	0.08
	(-6.7-4.1)	(-10.9– 0.02)		(1.8)	(1.6)		(1.4)	(2.6)		(1.4)	(2.4)	
MTPM	1.15	1.03	0.8	1.57	1.94	0.3	1.46	2.4	0.02	1.53	2.07	0.19
	(0.2–7.1)	(0.6–13.4)		(1.8)	(1.7)		(1.1)	(3.0)		(1.2)	(2.7)	

Cement grade: A = optimal, B/C = less than optimal.

All results are presented in mm. 2 stems were in valgus position.

^**a**^ Mann-Whitney U-test.

^**b**^ Student t-test.

78% of the 114 stems had cementing technique of Barrack grade A. Regarding the 8 revised stems, 2 had been implanted using an inferior cementing technique and in another 2 cases the tip of the stem pointed dorsally. In the 4 remaining hips, 1 had been implanted in valgus and 1 in varus (Table 2).

Using templates corrected for magnification, the implanted stem prosthesis was smaller than template in only 2 of 87 cases analyzed; in 10, the sizes corresponded and in the remaining 75 the implanted stem was larger than what the template indicated.

### RSA

**Figure 3.**
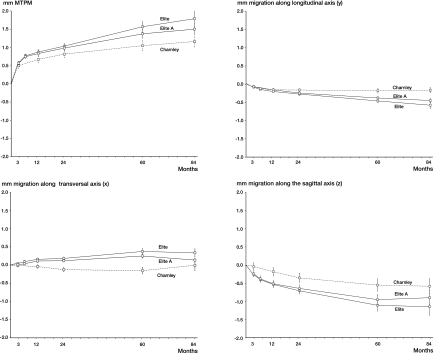
Migration pattern for the Elite-Plus stem in the present study and 40 Charnley monobloc stems from a previous study (önsten et al. 1995). All values are signed and in mm. Error bars indicate standard error. “Elite” includes all patients. “Elite A” includes only patients with cementation technique of grade A according to Barrack et al. (1992). A. Maximum total point motion (MTPM). B. Distal migration along the y-axis (a negative value indicates distal migration). C. Medial-lateral migration along the x-axis (a positive value indicates medial migration). D. Antero-posterior migration along the z-axis (a negative value indicates posterior migration).

RSA evaluation of the stem was not possible in 16 patients for technical reasons, leaving 98 stems for analysis. The number of examinations able to be analyzed was 95, 94, 95, 81, and 66 at 3 and 6 months and at 1, 2, 5 and 7 years, respectively. At the last follow up, 54 of the 98 stems had migrated over the level of accuracy in the medial direction and 15 laterally (> 0.2 mm). 74 stems had migrated distally (> 0.2 mm). 8 stems had migrated in the anterior direction and 69 in the posterior direction (> 0.5 mm). 95 of the 98 stems had migrated over the level of accuracy of the method in one or more of the directions (Figure 3).

The stems continued to sink; there was a difference in distal migration between the 5- and 7-year follow-ups (p = 0.006). However, there was no statistically significant difference in migration between the 5- and 7-year follow-ups in the dorsal or transversal direction of the remaining 63 stems that were available for RSA analysis (p = 0.1, Wilcoxon). At the last examination before revision, the 8 revised stems had an MTPM value of 3.2 mm (SD 4.4) as compared to 1.6 mm (SD 1.3) for the unrevised stems (p = 0.001).

There was no difference in stem migration (in any direction) between the 50 patients with a Hylamer cup and the 48 patients with an Enduron cup. The migration was increased in the medial and posterior directions for the 26 stems that were considered to be too small at the postoperative radiographic analysis (Table 2). This difference was not statistically significant, however (p = 0.2 and p = 0.08, respectively).

## Discussion

Contrary to the considerable number of socket problems encountered in our series of patients, which has been presented elsewhere (von Schewelov et al. 2005), the number of Elite-Plus stems that had to be revised due to aseptic loosening was not very alarming. The stem survival of 98% at 7 years and 92% at 10 years is similar to that reported for most current stem designs. However, 7 unrevised stems had minor or non-progressive radiographic signs of loosening, and these patients will therefore be followed on a continuous basis.

The migration of the stems appears to have stabilized after 5 years along the transverse (x-) and sagittal (z-) axes, but the slope of the curve indicates a slight but statistically significant distal migration along the longitudinal (y-) axis. 95 of the 98 stems had migrated along 1 or more of the axes above the level of accuracy for the method, the major part (69) in the posterior direction.

We found that migration along any of the axes was higher (p < 0.05) if the cementing technique was inferior (Barrack B and C). There was also a tendency of medial and posterior migration if the size of the implant was considered too small. Stem position, wear of the acetabular component, or whether a Hylamer or Enduron cup was implanted had no influence on stem migration.

There were more stem revisions in patients with a Hylamer socket (6/58 vs. 2/56) and the revision rate would have been halved if cases with a Hylamer cup had been excluded. It is likely that an abnormally high number of wear particles finds its way down the proximal part of the stem, inducing osteolysis (Anthony et al. 1990). Although the stem is fixed in the distal part, the loss of proximal support increases the risk of stem failure, which may cause the surgeon to revise the stem en passant. Indeed, 3 of the revised stems in our study were fixed at revision.

The migration of the Elite-Plus stem was higher than the migration of 40 conventional Charnley stems followed for 7 years (önsten et al. 1995). The difference in medial (p < 0.001), transverse (p = 0.02), longitudinal (p = 0.02), and MTPM (p = 0.08) migration was already obvious at 2 years (Student’s test). The inclusion criteria for the 2 study groups were the same and they were similar regarding age, weight, sex, and side operated (p = 0.3, p = 0.8, p = 0.07, and p = 0.3, respectively; chi-square). The surgical technique and the bone cement used were also the same in the 2 studies, both of which were performed at our institution. The digital RSA analysis in the present study may have had a slightly better precision than our previous method, but otherwise there was no difference in methodology.

Table 3 gives overview of relevant studies of the Charnley Elite Plus stem. Alfaro-Adrian et al. (2001) presented the 2-year RSA result of 19 Elite-Plus stems in a study comparing migration between the Elite and Exeter designs. Not surprisingly, it was found that the polished Exeter stem subsided more rapidly than the Elite stem. It was also observed that the Elite-Plus head migrated twice as much in the posterior direction than the Exeter head. 4 of the 19 stems that had a high rate of posterior head migration were later shown to have failed (2 were revised and 2 were radiographic failures) (Hauptfleisch et al. 2006). It was later pointed out that 3 of these 4 implants had an “inadequate cement mantle” (Porter and Derbyshire 2003, Isaac 2006).

**Table 3.** An overview of relevant studies of the Charnley Elite-Plus stem

Authors	Year	No. of cases	Length of follow-up	Type of cement	Revised stems ^**b**^	Loose on radiography incl. revisions
Alfaro-Adrian et al. (2001)	2001	19 RSA	2 years	Low-viscosity	n.s.	n.s.
Kalairajaha et al. (2004)	2004	200	5 years	High-viscosity	0	3 (1%)
				(Palacos R with Genta.)		
Rowsell et al. (2005)	2005	268	4.5 years (mean)	Both high- and low-viscosity	5 (2%)	17
Walton et al. (2005)	2005	168	6.4 years (mean)	Both high- and low-viscosity	9 (5%)	52 (31%)
Hauptfleisch et al. (2006)^**a**^	2006	118	9 years (mean)	Low-viscosity	12 (10%)	24 (23%)
Kim et al. (2007)	2007	194	11.2 years (mean)	Medium-viscosity	2	3 (2%)
				(Simplex-P)		
Derbyshire and Porter (2007)	2007	29 (25 RSA)	3 years (mean)	High-viscosity (CMW 2)	n.s.	n.s.
Present study	2008	114 (RSA)	mean 6.5	High-viscosity		
			(2–9.5)	(Palacos R with Genta.)	8 (7%)	15 (13%)

^**a**^ Including the 19 cases presented by Alfaro-Adrian et al. (2001)

^**b**^ Revisions for aseptic loosening.

n.s.: not stated.

In a recent RSA study, Derbyshire and Porter (2007) found that the Elite-Plus stems continued to subside during the 3-year observation period. They also noted that the stems continued to rotate internally and to migrate in the posterior direction. However, these latter movements were not statistically significant. Due to the lack of tantalum markers on the stems in our study, we could not evaluate rotation, but there was no statistically significant change in posterior migration of the femoral head between 5 and 7 years. The level of migration in the posterior direction was similar in the study by Derbyshire and Porter and in the present study, but there was less migration along the transverse axis in our study. A slow distal migration during the first 3 years shown by Derbyshire and Porter (2007) was confirmed in our study and continued up to 7 years.

In the literature, 0–10% of the Elite-Plus stems have been revised after 3–10 years and the rate of radiographically loose stems has varied between 1% and 31% (Alfaro-Adrian et al. 2001, Kim et al 2001, Kalairajah et al. 2004, Rowsell et al. 2005, Walton et al. 2005, Hauptfleisch et al. 2006). Our results with the Charnley Elite-Plus stem lie in the middle between these extremes. Should the prosthesis, the surgical technique, or the type of bone cement be blamed for these poor results? The most disappointing result was from a series using low-viscosity cement throughout (Hauptfleisch et al. 2006) and the best results have been from studies in which only high-viscosity cement was used (Kalairajah et al 2004, Derbyshire and Porter 2007). These results thus confirm earlier reports, e.g. from the Norwegian Arthroplasty Register, of an increased failure rate in cases where low-viscosity cement has been used (Espehaug et al. 2002).

Cementing technique is apparently also of importance. Cementing of grades B and C (22 of 98 stems) was associated with increased migration in our study. Cementing quality was not accounted for in the study by Hauptfleisch et al. (2006), but using both high- and low-viscosity cements, Walton et al. (2005) found that the number of revisions was significantly higher if Elite-Plus stems had had a deficient cement mantle and when low-viscosity cement had been used.

The Swedish Hip Arthroplasty Register (2007) reported a survival rate due to aseptic loosening at 7 years of 97%, and 94% at 10 years, for all cemented implants. Our survival rate of 92% for the Elite-Plus stem is similar to this figure.

The number of revisions in our study is not alarming compared to other cemented designs in use, and clinical and radiographic performance of the stem was found to be “normal”. However, the increased migration of the Elite-Plus stem as compared to the Charnley stem is presumably due the Elite-Plus stem being more sensitive to implantation technique and cement used. We therefore suggest that patients with a Charnley Elite-Plus stem and radiographic signs of inferior implantation technique should be followed, especially if low-viscosity cement has been used.
